# Increased Toll-Like Receptor-4 Signalling in Breast Tissue of High Fibroglandular Density

**DOI:** 10.1007/s10911-025-09593-5

**Published:** 2026-01-07

**Authors:** Hanieh Heydarlou, Leigh J. Hodson, Pallave Dasari, Eric Smith, Wendy V. Ingman

**Affiliations:** 1 School of Medicine, Adelaide University, The Queen Elizabeth Hospital, DX465702, 28 Woodville Rd, Woodville, Adelaide, 5011 Australia; 2 Robinson Research Institute, Adelaide University, Adelaide, Australia; 3 Medical Oncology, Basil Hetzel Institute, The Queen Elizabeth Hospital, Woodville South, SA 5011 Australia

**Keywords:** Mammographic density, Toll-like receptors, Inflammation, Cytokines, Breast cancer risk

## Abstract

High mammographic breast density relates to the abundance of fibroglandular tissue in comparison to fatty tissue in the breast and is associated with increased breast cancer risk. Chronic low-level inflammation has been implicated as a driver of high density and cancer risk, however little is understood of the underlying cause of inflammation. This research aimed to investigate the role of the innate immune recognition receptor toll-like receptor-4 (TLR4) in inflammation associated with high fibroglandular density. Immunohistochemical analysis was performed on paired breast tissue samples of high and low fibroglandular density tissue (*n* = 22 pairs) to investigate the expression of TLR4, TLR4 agonists lipopolysaccharide (LPS) and damage response protein high-mobility group protein 1 (HMGB1), as well as activation of downstream mediators myeloid differentiation primary response 88 (MYD88) and nuclear factor kappa B (NFKB). Mammary epithelial cell organoids (*n* = 5) were cultured in vitro with LPS to investigate the expression of genes associated with inflammation. TLR4 was primarily expressed in basal epithelial cells, stromal macrophages, and some expression was detected in luminal epithelial cells. Increased expression of TLR4, MYD88, NFKB, and HMGB1 was observed in epithelial cells in high fibroglandular density tissue. There was increased expression of the genes encoding inflammatory cytokines tumour necrosis factor alpha (TNFA) and C-C motif ligand 2 (CCL2) in mammary epithelial cell organoids treated with LPS. The TLR4 signalling pathway may be a mediator of local breast inflammation associated with regions of high breast density and the damage response protein HMGB1 may be a trigger for TLR4 activation.

## Background

Mammographic density refers to the radiological appearance of breast tissue on an X-ray mammogram and is an independent risk factor for breast cancer [1]. From a biological perspective, mammographic density relates to the relative abundance of stroma and epithelium, known collectively as fibroglandular tissue, compared to the abundance of fatty adipose tissue [2]. Mammary epithelial cells comprise two main types of luminal and basal epithelial cells. Despite the increase in total epithelial content, the ratio of basal to luminal cells remained unchanged [3]. Areas of breast that are predominantly comprised of fibroglandular tissue appear white on a mammogram and are considered dense, areas that are predominantly fatty appear dark and are considered non-dense. Women with extremely dense breasts have a 4- to 6-fold increased risk of breast cancer compared to women with breasts classified as mostly fatty, when adjusted for age and body mass index [4, 5]. High mammographic density can also mask cancers on a mammogram and delay breast cancer detection [6]. Although mammographic density is a well-established risk factor for breast cancer, the underlying biological mechanisms that lead to high mammographic density are still under investigation.

A challenge for biological research in mammographic density is in obtaining breast tissue samples of known density. Breast tissue is highly heterogeneous, even within a breast with very high mammographic density there are regions of low density, and vice versa. This difficulty can be overcome by using surgically excised breast tissue which is sliced and X-rayed to guide biopsies of high and low density regions from the same individual [7]. High and low density tissue samples from the same individual can be statistically analysed as paired samples, which has been a powerful approach to understand the biological changes associated with high density. This paired sample approach has shown that high mammographic density is associated with increased relative abundance of epithelium, stromal fibroblasts and collagen, and reduced relative abundance of adipocytes [7], and that fibroglandular density can be used as a surrogate marker for mammographic density [3].

There is growing evidence that high mammographic density is linked with a pro-inflammatory microenvironment which may act to increase breast cancer risk. Increased abundance of macrophages, dendritic cells, CD4 + T cells, B cells and vimentin positive immune cells is observed in tissue with high mammographic density [3, 8]. Vimentin is expressed on all stromal tissues and in breast cancer, correlated with poor survival rate [9]. In women with high mammographic density, inflammatory mediators such as cyclooxygenase-2 (COX2), cytokines including tumour necrosis factor alpha (TNFA), interleukin 6 (IL-6), and chemokines like C-C motif ligand 2 (CCL2) are increased while, anti-inflammatory transforming growth factor beta (TGFB) signalling is reduced [10–13]. Pro-inflammatory signalling may be a driver of mammographic density and the associated increased risk of cancer as constitutive expression of CCL2 in the mammary gland of a mouse model increased fibroglandular density, local inflammation, and mammary cancer susceptibility [13]. However, it is unclear what causes the local inflammation associated with dense breast tissue.

Pattern Recognition Receptors (PRRs) are components of the innate immune system that recognise pathogen-associated molecular patterns (PAMPs) present in microorganisms and damage-associated molecular patterns (DAMPs) released from dying or damaged cells. Activation of PRRs leads to activation of transcription factors such as nuclear factor kappa B (NFKB), resulting in expression of pro-inflammatory cytokines, adhesion molecules, and extracellular matrix regulators that promote infiltration and activity of immune cells in the local tissue microenvironment [14]. Amongst the PRR family, toll-like receptor 4 (TLR4) has the largest number of diverse ligands from endogenous DAMPs and exogenous PAMPs [15, 16] and is expressed on both epithelial cells and macrophages in breast tissue [17].

TLR4 activation generates a potent immune response, and this has been most commonly studied using the TLR4 agonist lipopolysaccharide (LPS), which is an immunostimulatory component of bacterial cell walls [18]. The breast is not a sterile tissue, invading pathogens can enter the epithelial ducts via the nipple [19] which could potentially be a trigger for TLR4-mediated inflammation. In addition, it has also been demonstrated that under sterile conditions, TLR4 can be activated and contribute to the development of antigen-specific adaptive immunity [20]. Endogenous activators of TLR4 are DAMPs such as high-mobility group protein 1 (HMGB1), a damage-associated nuclear protein released during tissue injury. Upon activation, MYD88, the protein adaptor, is recruited into the tail domain of TLR4 to initiate the signalling pathway inducing the expression of genes that mediate inflammatory responses.

Activation of the TLR4 signalling pathway has been intensively studied using lipopolysaccharide (LPS) as a model agonist. It is well-established that the TLR4 pathway induces the release of proinflammatory cytokines [18]. Commonly, TLR4, localised on the cell surface is responsible for recognising LPS; however, LPS can occasionally enter the cytosol via CD14. CD14 is a co-receptor for TLR4, which binds to LPS. CD14 can also mediate the internalisation of LPS into the cytosol in a TLR4-independent manner. In the cytosol, LPS activates caspase-4/5, leading to noncanonical inflammation cell lysis and pyroptosis [21, 22]. Despite these TLR4 independent effects, LPS remains the predominant TLR4 agonist that has been applied in research, as alternative agonists such as HMGB1 are often complex, variable, and lack receptor specificity. For example, HMGB1 binds to both TLR4 and the Receptor for Advanced Glycation Endproducts (RAGE) [23].

The aim of this study was to investigate the TLR4 signalling pathway as a potential upstream mediator of inflammation in dense breast tissue. Using paired samples of high and low fibroglandular density tissue from the same patient, we demonstrate that expression of a number of the components of the TLR4 signalling pathway are increased in breast tissue with high fibroglandular density. LPS, a pathogen-derived TLR4 agonist, was present in the breast tissue but not altered by fibroglandular density, however the endogenous agonist HMGB1 was increased in high fibroglandular density tissue. Activation of TLR4 resulted in increased expression of genes encoding inflammatory cytokines, including TNFA and CCL2 in mammary organoid cultures. Collectively, these findings support the notion that TLR4 may be a component of immune signalling that leads to chronic inflammation in high mammographic density tissue. Furthermore, this study suggests that the stimuli for TLR4 are likely to be endogenous damage response proteins rather than external invading pathogens.

## Materials and Methods

### Breast Tissue Collection

Central Adelaide Local Health Network Human Ethics Research Committee approved this study (TQEH Ethics Approval #2011120). Participants aged 18 to 75, who underwent cosmetic reduction, prophylactic mammoplasty, and mastectomy for breast cancer removal, generously donated tissue. Participants who were pregnant or had undergone chemotherapy, as well as those with a large breast tumour or multiple tumours, were excluded from the study. Breast tissue was collected directly from the surgical theatre and used for histology and in vitro culture studies.

### Breast Tissue Histology

For histology studies, a portion of breast tissue samples were dissected into small portions and fixed in 4% paraformaldehyde (Sigma Aldrich, St. Louis, MO, USA; Cat#P6148) for 7 days at 4 °C. Tissue were processed using a Leica TP1020 Tissue Processor (Leica Microsystems, Wetzlar, Germany) and embedded in paraffin wax. Sections of 5 μm thickness were cut using a Leica Rotary Microtome (Leica Microsystems), and slides were incubated overnight at 37 °C to promote tissue adhesion.

### Breast Tissue Processing for Mammary Epithelial Organoids

For mammary epithelial organoids, fresh breast tissue had excess fat removed prior to being finely minced and enzymatically digested with 100 U/mL of Hyaluronidase (Sigma Aldrich; Cat# H3506) and 480 U/mL of Collagenase (Sigma Aldrich; Cat# C0130) in Advanced DMEM/F12 medium (Life Technologies, Carlsbad, CA, USA; Cat# 12491015) containing 2.5 mg/mL Fungizone (Life Technologies; Cat# 15290018), 100 U/mL Penicillin/Streptomycin (Life Technologies; Cat# 15240062), 10 mM HEPES buffer (Life Technologies; Cat# 15630080), and 100 µg/mL L-Glutamine (Life Technologies; Cat# 25030081). The digestion flask was placed in a 37 °C shaking water bath for 16 h to separate the stromal and epithelial components of the tissue. The digested tissue was diluted with DMEM media at least 1:3 and centrifuged at 80 x g for 1 min at a moderate brake to separate the digested tissue into the top lipid layer, middle layer of stromal single cells, and bottom pellet containing mammary epithelial organoids and undigested breast tissue pieces. These three layers were harvested for further processing.

To isolate mammary epithelial organoids, the bottom pellet containing mammary epithelial cells and undigested tissue was transferred to fresh 50 mL Falcon tubes. DMEM media was added to wash and mix the pellet before sitting the tubes for 30–60 s for gravity settling of undigested tissue and then harvesting the supernatant containing mammary epithelial organoids to a separate tube. Mammary epithelial organoids were washed again with DMEM/F12 media and isolated from the supernatant through gravity settling for four minutes; this step was repeated to wash the organoids until the supernatant was clear. After organoids were verified via microscopic analysis, organoids were centrifuged at 480 x g for 1 min, supernatant removed, and resuspended in Advanced DMEM/F12 medium supplemented with 50% heat-inactivated fetal bovine serum (FBS; Gibco, Thermo Fisher Scientific, Cat#10091-148), 6% Dimethyl sulfoxide (DMSO; Sigma-Aldrich, Cat#34869), Penicillin/Streptomycin, Fungizone, glutamine, and Fungizone, and stored in liquid nitrogen.

### Haematoxylin and Eosin Staining

Formalin-fixed-paraffin-embedded (FFPE) breast tissue blocks obtained were subjected to hematoxylin and eosin (H&E) staining. First, breast tissue sections were dewaxed using 3 different containers of xylene (Merck Millipore, Darmstadt, Germany; Cat#108298), each for 5 min, secondly, they were rehydrated in gradual dilutions of ethanol (2 × 100%, 1 × 90%, 1 × 70% and 1 × 50%), each for 3 min, followed by 3 min in distilled water. Tissue sections were stained with hematoxylin (Sigma Aldrich, St Louis, MO, USA; Cat#HHS16) and eosin (Sigma Aldrich, St Louis USA; Cat#318906), respectively for 30 and 10 s. Sections were then dehydrated through a gradual increase in ethanol concentration (1 × 90%, 2 × 100%) and cleared with 2 different containers of xylene for 5 min. Finally, slides were coverslips with mounting media (Proscitech, Kirwan, QLD, Australia; Cat#IM022) and left to dry and further analysis.

### Histological Index of Mammographic Density

Fibroglandular density was assessed as a surrogate indicator for mammographic density as described previously [24, 25]. Briefly, a panel of three scientists (H.H., L.H., W.I.) observed haematoxylin and eosin-stained breast tissue sections on a semi-quantitative scale. The assessment was based on the relative abundance of fibroglandular tissue and adipose tissue in the section. For each tissue block, one tissue section was assessed, and the panel reached a consensus on density through discussion. Higher density scores were assigned to sections containing a greater percentage of stroma and epithelium. The classification scale ranged from 1 to 5, where 1 represented 0–10%, 2 represented 10–25%, 3 represented 25–50%, 4 represented 50–75%, and 5 represented > 75% of fibroglandular tissue [26]. This method has demonstrated a reliable correlation with mammographic density determined by X-ray [3]. Tissue scoring between 1 and 2 was classified as low mammographic density, while tissue scoring between 4 and 5 was classified as high mammographic density. Tissue scoring 3 was not classified as low or high and was not utilised in this study [26].

### Immunohistochemistry Staining

The expression of TLR4, MYD88, NFKB, LPS and HMGB1 were assessed through immunohistochemistry. As described previously, dewaxing and hydrating tissue were applied. Then, antigen retrieval was performed using the high pH retrieval solution (DAKO, Cat#K8004) on paired high and low mammographic density tissue. Later, the tissue was incubated with endogenous peroxidase block for 10 min at room temperature. The sections were washed three times for three minutes in 1x PBS buffer, and then the mouse monoclonal to TLR4 with the concentration of 1/50 (Abcam, Cat#ab22048), 1/500 rabbit monoclonal to MYD88 (Abcam, Cat#ab133739), 1/1000 rabbit polyclonal to NFKB p65 (Abcam, Cat#ab16502), 1/300 mouse monoclonal to E.coli LPS (Abcam, Cat#AB35654), 1/400 rabbit monoclonal to HMGB1 (Abcam, Cat#AB79823 were applied and incubated in a humid chamber, overnight at 4 degrees Celsius. Later, the sections were treated with an HRP-conjugated secondary antibody (Agilent Technologies, Singapore; Cat#K346811-2), after 45 min, diaminobenzidine (DAB) (Agilent Technologies; Cat#K346811-2) was added to the slides and incubated for 10 min at room temperature. The slides were then properly washed in distilled water, counterstained with hematoxylin, dehydrated in different percentages of ethanol from 90% to 100%, and 2 changes of xylene to be coversliped with a mounting medium.

### Quantification of Immunohistochemistry Images

All stained immunohistochemistry slides were digitally scanned at a magnification of 40x and resolution of 0.23 μm using the NanoZoomer Scanner 2.0-HT (The University of Adelaide, Adelaide Microscopy). The intensity of the staining was quantified using ImageJ (Schneider, C. A., Rasband, W. S., & Eliceiri, K. W. 2022. ImageJ, Version 1.53). Colour deconvolution was applied to separate the hematoxylin and DAB staining components. In ImageJ, the pixel intensity values for any colour range from 0 to 255, where 0 corresponds to the darkest shade and 255 displays the lightest shade of the colour. For quantitative assessment, multiple random regions of both epithelium and adjacent stromal tissue were identified on each histological section. Within each region, the intensity of staining was measured using a fixed threshold setting, which was applied uniformly across all pairs. For each section, the measurements from all selected regions were averaged to yield a single representative value. The mean intensity value from each slide was taken for statistical analysis.

### Immunofluorescence Staining

The localisation of TLR4 on epithelium and stroma was investigated by immunofluorescence staining using a mouse monoclonal antibody to TLR4 (Abcam, Cat#ab22048) at a dilution of 1/50, followed by secondary goat anti-mouse 647 Alexa fluor conjugated antibody (Invitrogen, Lot#WB316325). Keratin 17 (KRT14) 594 Alexa fluor conjugated antibody (Santa Cruz Biotechnology, Sc-53253#LotDo822) at a dilution of 1:100, and Keratin 19 (KRT19) 488 Alexa fluor conjugated antibody (Invitrogen, eBiosciense #Lot 22199998) at a dilution of 1:500, were used to identify basal and luminal epithelial cells respectively. Co-localisation of CD68-positive macrophages with TLR4 expression and LPS-positive cells was also determined using the antibodies mentioned above. To investigate cellular location of HMGB1, immunofluorescence staining was performed using HMGB1 antibody (Abcam, Cat#AB79823) and followed by secondary goat anti-rabbit 568 Alexa fluor conjugated antibody (Invitrogen, Cat#A-11011), on high and low density tissue. Briefly, breast tissue sections were deparaffinized and hydrated, followed by antigen retrieval. Blocking non-specific binding was applied by incubating tissue with 10% goat serum in BSA for 30 min at room temperature, plus TrueBlack Lipofuscin Autofluorescence Quencher (Biotium, Fremont, CA) for 45 s. Primary and conjugated antibodies were incubated overnight at 4 °C, and the secondary antibody was incubated for 1 h at room temperature. After each step slides were washed 3 times for 3 min with PBS. Finally, slides were stained with DAPI for 5 min and coverslipped using fluorescent mounting media. Immunofluorescent images were captured using a Zeiss LSM 900 confocal microscope (Carl Zeiss AG, Oberkochen, Germany). Where comparison between high and low fibroglandular density was required, the fluorescence intensity setting was kept constant throughout the entire imaging process. All secondary antibody was applied at a 1/500 dilution.

### 3D Culture of Human Epithelial Organoids

After collecting from liquid nitrogen, organoids were suspended in Human Mammary Epithelial Cell Basal Medium (Life Technologies; Cat# M171500), supplemented with 1X Mammary Epithelial Growth Supplement (MEGS) (Life Technologies; Cat# S0155). Next, cells were centrifuged at 400 x g for 5 min, and the pellet was resuspended in 1 mL media. Working quickly on ice, organoids were generated using the dome approach, where cell suspensions were mixed well with Matrigel (BD, Growth Factor Reduced, Phenol Red-Free #356231), at a 1:2 ratio. One drop of 25 µL Matrigel-cell mixture with the density of 5× 10⁴ cell was placed in the centre of a prewarmed 24-well suspension sterile cell culture plate (Greiner Bio-One; Cat# 662102). The plate was incubated at 37 °C for 20 min to allow Matrigel to solidify. Once the Matrigel polymerised, 500 µl of complete Medium, supplemented with 10 µM of Y-27,632 dihydrochloride, Rho kinase inhibitor (Abcam, Cat# ab120129), was carefully added to the well on top of the dome. The culture Medium was changed every 3–4 days and 3 days after initial seeding, Y-27,632 was removed from the Medium. Cells were cultured for around 10 days to allow organoids to stabilise and grow before treatment.

3D organoid cultures were incubated with 10 mL of LPS (Sigma-Aldrich, #L2630) at 37 °C, 5% CO_2_ for 24 h. After gently aspirating the Medium, cold Cell Recovery Solution (Bio-Strategy, #Cat BDAA354253) was added into wells, droplets were then mechanically disassociated with a cell scraper. Once the organoids were dislodged, they were transferred into sterile tubes and incubated on ice for 2 h to fully dissolve the Matrigel.

### Quantification of Gene Expression

Total RNA was isolated using RNeasy mini kit (QIAGEN, Australia; cat#74104) as per the manufacturer‘s instructions. For complete DNA removal, RNase-Free DNase Set (QIAGEN, Australia; cat#79254) was used. The concentration of purified RNA was measured by absorbance at 260 nm relative to absorbance at 280 nm using Spectrophotometer ND 1000 Nanodrop with program ND-1000 V3.7.1. Later the appropriate concentration of RNA was reverenced transcript to cDNA using Moloney murine leukemia virus (MMLV) enzyme from iScript synthesis kit (Bio-Rad technologies; Cat#1708890). The 20 µL reaction contains 4 µL iScript reaction Mix, 1µL iScript reverse transcriptase, 2 µL isolated RNA, and 13 µL nuclease-free water. The complete reaction mix was incubated in the thermal cycler as follows 5 min at 25 °C, 20 min at 46 °C, and finally 1 min at 95 °C.

Real-Time PCR was conducted to quantify gene expression of *TLR4*,* MYD88*,* NFKB*,* HMGB1*,* TNFA*,* TGFB1*,* CCL2*,* COX2* and *VIM*. The 10 µL reaction contained 5 µL of SYBR green master mix (Bio-Rad), 0.5 µL of each forward and reverse primers, 2 µL of cDNA, and 2 µL of nuclease-free water. Reactions were run on the thermocycler machine (Applied Biosystems ViiA 7) with following thermal cycling protocol; DNA denaturation at 95 °C for 3 min, amplification with 40 cycles of 15 s at 95 °C, 60 s at 60 °C, and 72 °C for 30 s. Each run contains no template control of each gene. Beta-Actin was used as a reference gene. Real-time PCR data were analysed using the ΔΔCt method, comparing the expression levels of target genes relative to control groups and normalised to the reference gene.

### Statistical Analysis

Statistical analysis was conducted using SPSS software (IBM.SPSS statistics, Chicago, IL, USA) version 28.01.1, and GraphPad Prism 8 (GraphPad software Inc, San Diego, USA) version 10.1.0. when *p* < 0.05, also showed as an asterisk (*), data are considered significantly different from the control, or data between low and high fibroglandular density are significantly different. All data in this study are presented as mean ± SEM (standard error of mean). The quantification of the paired sample from the immunohistochemistry study was performed by a non-parametric Wilcoxon test. The qPCR RT data were assessed based on fold change. mRNA expression level was analysed, using 2-way ANOVA with Šídák’s multiple comparisons test.

## Results

A total of 44 FFPE tissue blocks comprising paired high and low fibroglandular density sections from 22 participants were analysed. In addition, primary mammary epithelial cells were isolated from a separate group of five participants for the in vitro study. Demographic characteristics of all participants are summarised in Tables 1 and 2 for FFPE and epithelium organoid samples, respectively.

**Table 1:** Demographic Characteristics of the Study Population for FFPE Samples

Variables	Number or Mean
Age (year)	44.27 (range 18-61)
BMI (Kg/m^2^)	29.37 (range 20.2-53.26)
History of benign breast disease	
Yes	4
No	18
Breast cancer-related gene mutation	
None	21
BRCA1/2	1
Family history of breast cancer	
Yes	9
No	13
Pregnancy	
Yes	19
No	3
Age at First Childbirth (year)	27.22 (range 16-41)
HRT^*^	
Yes	7
No	15

* Hormonal Replacement Therapy

**Table 2:** Demographic Characteristics of the Study Population for Epithelial Organoid Samples

Variables	Number or Mean
Age (year)	44.60 (range 30-58)
BMI (Kg/m^2^)	30.20 (range 22.25-37.72)
History of benign breast disease	
Yes	0
No	5
Breast cancer-related gene mutation	
None	4
BRCA1/2	1
Family history of breast cancer	
Yes	2
No	3
Pregnancy	
Yes	4
No	1
Age at First Childbirth (year)	27.75 (range 17-33)
HRT^*^	
Yes	2
No	3

* Hormonal Replacement Therapy

### Increased Expression of TLR4 Signalling Components in Breast Tissue Samples with High Fibroglandular Density

**Fig. 1:**
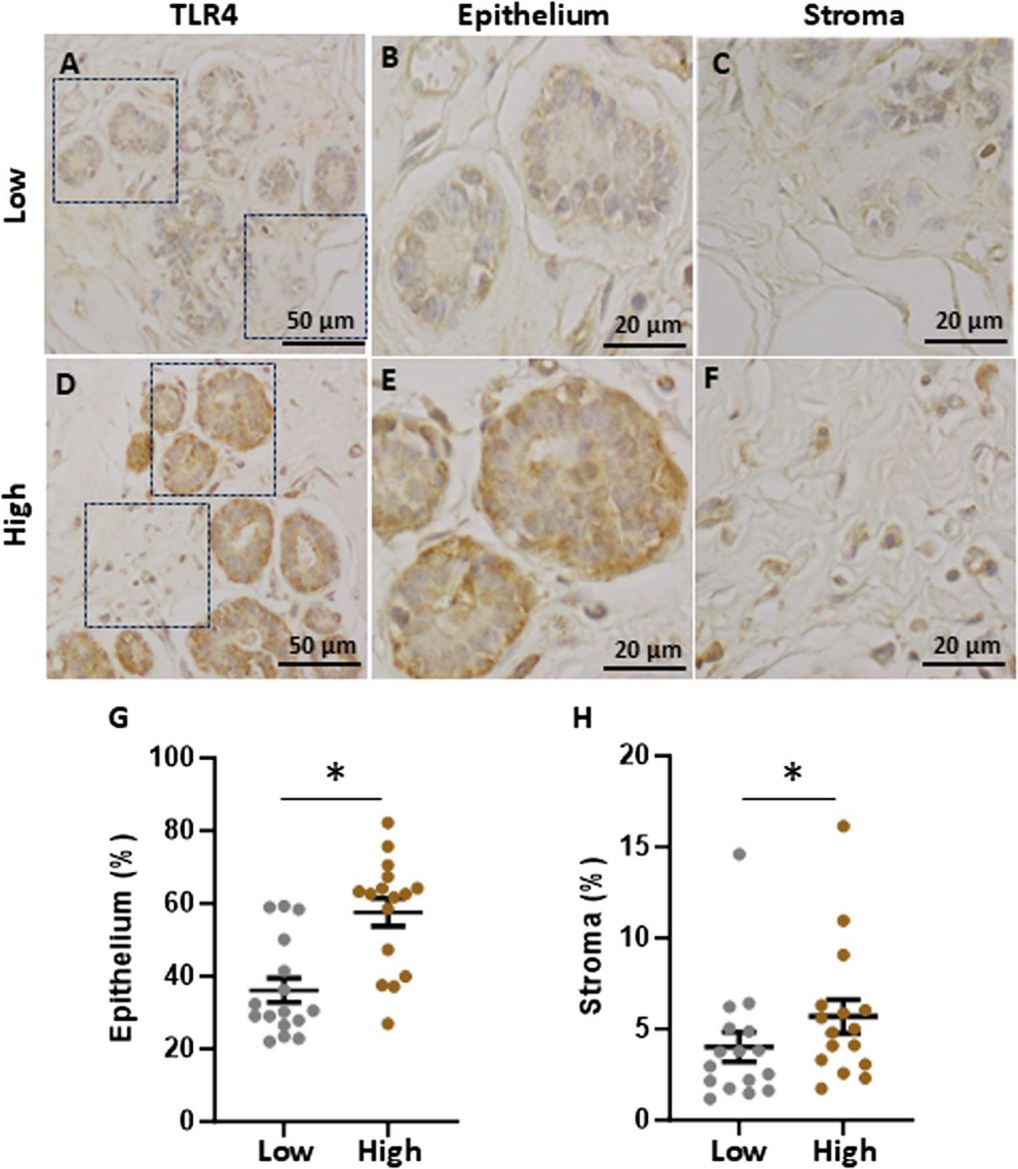
TLR4 expression in high and low fibroglandular density paired breast tissue samples. **A** and **D**: Representative immunohistochemical staining of TLR4 in low (**A**) and high (**D**) fibroglandular density breast tissue. TLR4 staining on epithelium (**B**) and stromal (**C**) region of low mammographic density. Staining of TLR4 in the epithelial (**E**) and stromal (**F**) compartments of breast tissue sections with high fibroglandular density. All zoomed-in images were captured at 100× magnification. The intensity of the TLR4 staining within epithelium (**G**) and stroma (**H**) was qualified between high and low fibroglandular density for each sample. Data were evaluated using paired samples and analysed with the nonparametric Wilcoxon test (*n* = 16); results are presented as mean ± standard error of the mean (SEM). Statistical significance indicated by * for *P* < 0.05

Immunohistochemistry staining demonstrated the presence of TLR4 in epithelium and stroma of breast tissue from high and low fibroglandular density (Fig. 1). Quantitative analysis showed increased expression of TLR4 in both epithelium (*P* < 0.001) and stroma (*P* = 0.026) of high fibroglandular density sections compared to the low fibroglandular density paired sample. Negative controls, prepared without the primary antibody, showed no detectable staining. Epithelial cells exhibited higher expression of TLR4 compared to stroma.

**Fig. 2:**
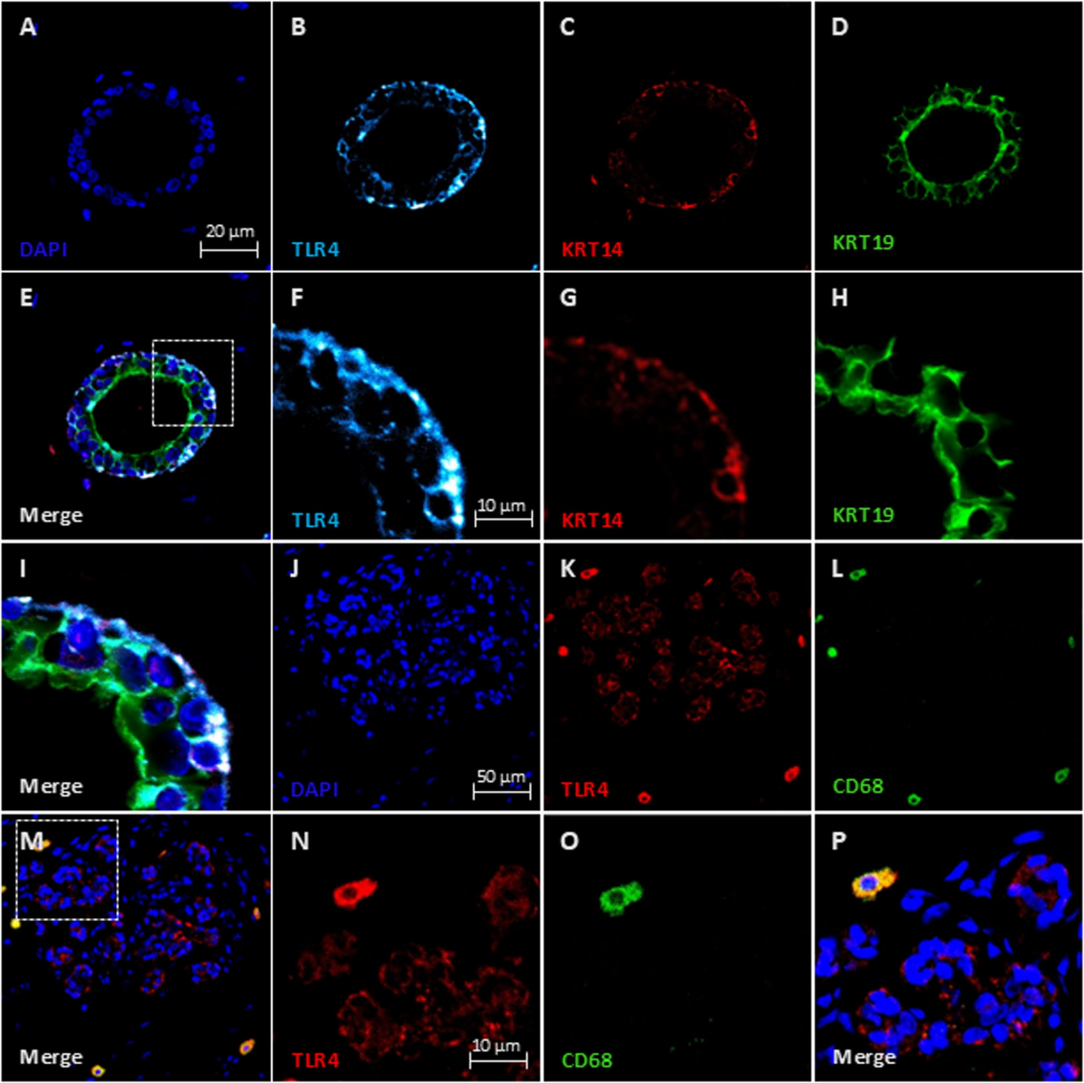
Epithelial cell types expressing TLR4 in high and low fibroglandular density samples. Representative immunofluorescent staining of breast tissue showing expression of TLR4 (**B** and **F**, light blue; K and N, red), Keratin 14 (KRT14) basal epithelial cells with red 594 Alexa fluor secondary (**C** and **G**, red) Keratin 19 (KRT19) luminal epithelial cells with green 488 Alexa fluor secondary (**D** and **H**, green), CD68-positive macrophages (green; **L** and **O**), and DAPI nuclear staining (blue; **A**,**J**). Merged images (**E**, **I**) showing co-localisation of TLR4 (light blue) with basal KRT14 epithelial cells [17] and slightly with luminal KRT19 epithelial cells (green). Merged images in the stromal region (**M**, **O**) representing TLR4 staining [17] with co-localisation on the surface of CD68-positive macrophages (green). All images include low- and high-magnification views to demonstrate localisation within epithelial and stromal compartments

Immunofluorescence staining was performed to determine the phenotypes of cells expressing TLR4 (Fig. 2). This showed that TLR4 was primarily located on basal epithelial cells, with some expression also observed on luminal epithelial cells. In the stroma, TLR4 expression was detected in CD68-positive macrophages.

**Fig. 3:**
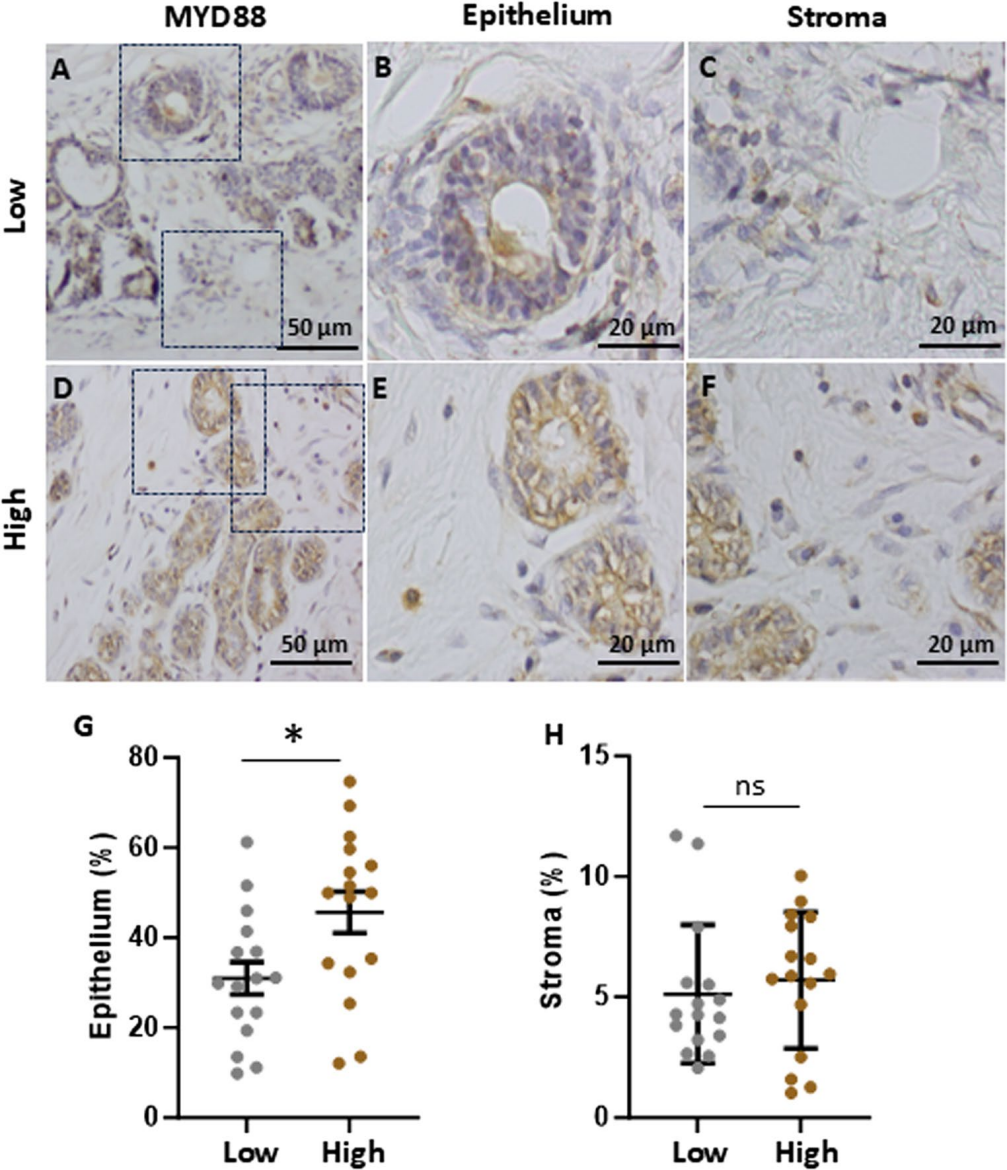
MYD88 expression in high and low fibroglandular density paired breast tissue samples. Representative immunohistochemical staining of MYD88 in low (**A**) and high (**D**) fibroglandular density human breast tissue. **B** and **E** display 100x magnification of epithelial cell staining on low (**B**) and high (**E**) fibroglandular density tissue. **C** and **F** show 100x magnification of stromal staining on low and high mammographic density tissue, respectively. The intensity of the MYD88 staining within the epithelium (**G**) and stroma (**H**) was qualified between high and low fibroglandular density for each sample. Data were evaluated using paired samples and analysed with the nonparametric Wilcoxon test (*n* = 16); results are presented as mean ± standard error of the mean (SEM). Statistical significance indicated by * for *P* < 0.05

**Fig. 4:**
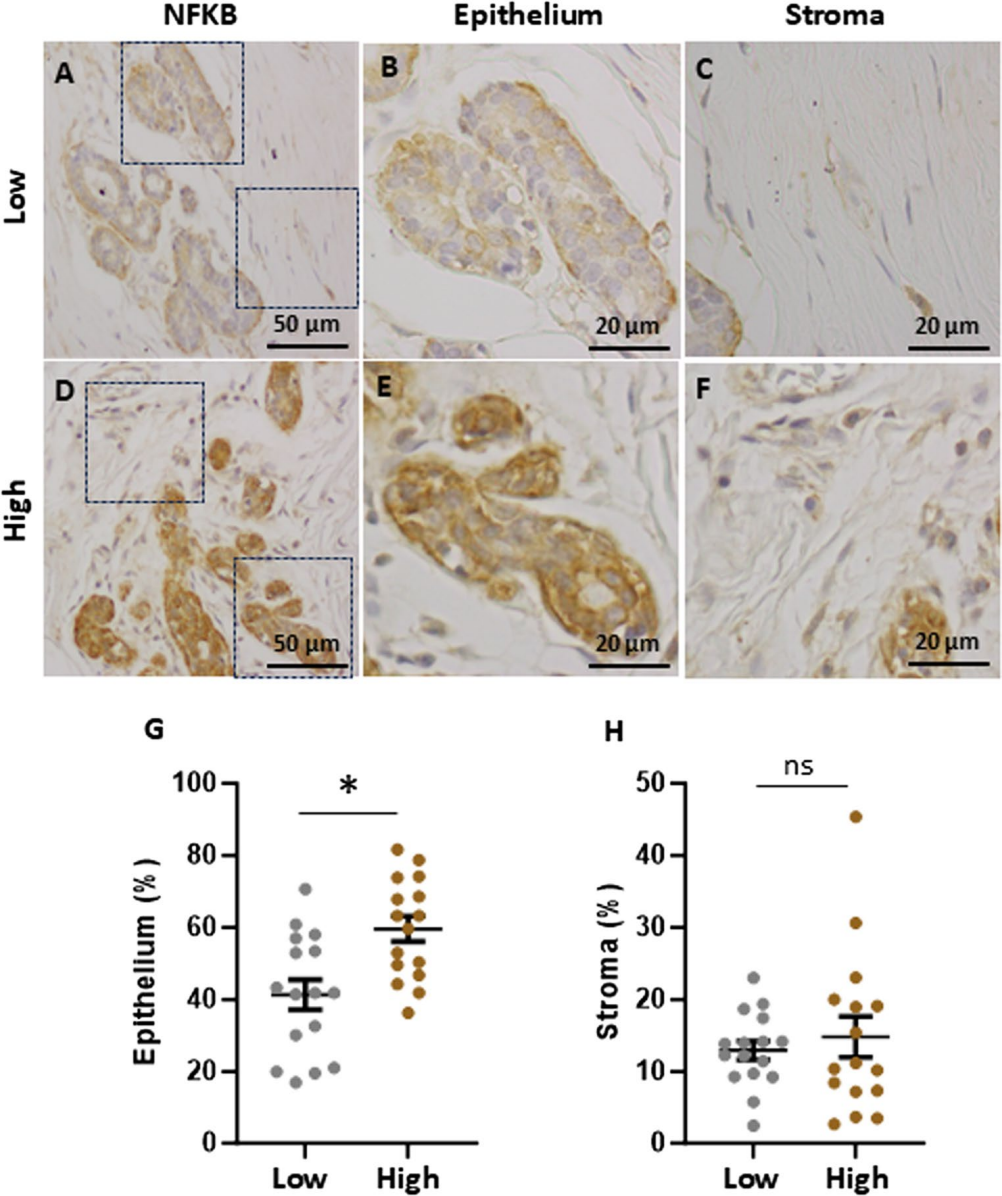
NFKB p65 expression in on high and low fibroglandular density paired breast tissue samples. Representative immunohistochemical staining of NFKB p65 in the epithelium of low (**A**) and high (**D**) fibroglandular density human breast tissue. NFKB p65 staining within epithelial cell layers of low (**B**) and high (**E**) fibroglandular density tissue. NFKB p65 staining on stromal region of low (**C**) and high (**F**) fibroglandular density tissues. The intensity of the NFKB staining within epithelium (**G**) and stroma (**H**) was qualified between high and low fibroglandular density for each sample. Data were evaluated using paired samples and analysed with the nonparametric Wilcoxon test (*n* = 16); results are presented as mean ± standard error of the mean (SEM). Statistical significance is indicated by * for *P* < 0.05

To investigate downstream components of TLR4 signalling, the adaptor protein MYD88 and the key transcription factor NFKB were examined by immunohistochemistry. Both MYD88 (Fig. 3) and NFKB p65 (Fig. 4) were observed in the epithelium and stroma of breast tissue samples. The intensity of staining for MYD88 and NFKB p65 was most notable in the epithelium with minor staining detected in the stroma. Notably, expression of both MYD88 and NFKB p65 was significantly higher in the epithelial cells from high fibroglandular density tissue compared to low-density tissue (*P value* of 0.023 and 0.007, respectively). No significant differences were observed in MYD88 or NFKB p65 expression in the stroma of high and low fibroglandular density tissue.

### Endogenous and Exogenous Stimuli of TLR4 in High and Low Mammographic Density

**Fig. 5:**
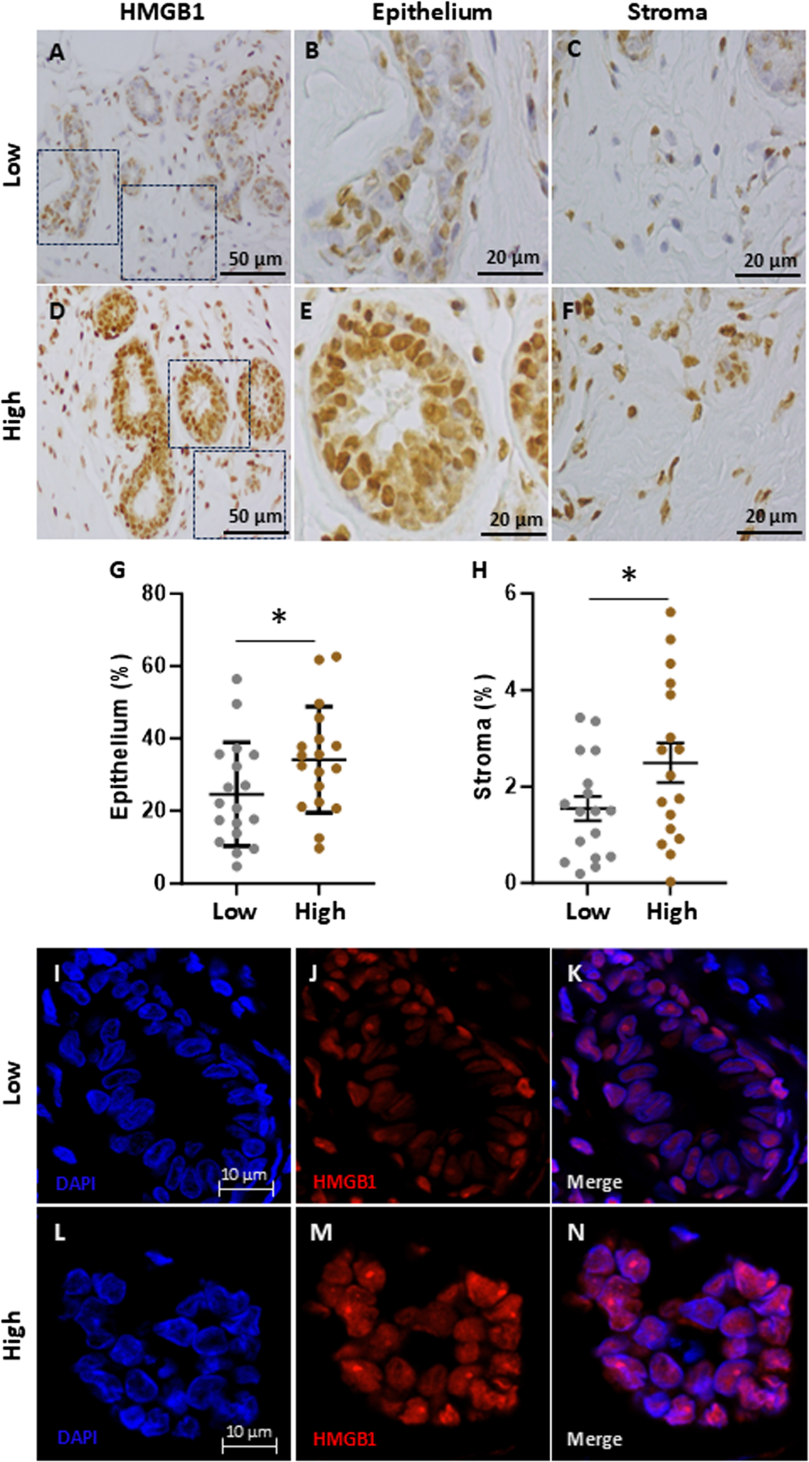
HMGB1 expression in high and low fibroglandular density paired breast tissue samples. HMGB1 immunohistochemistry staining of human breast tissue of low (**A**) and high (**D**) fibroglandular density. The intensity of the HMGB1 staining within epithelial cell was qualified between low (**B**) and high (**E**) fibroglandular density. Stromal region was investigated between low (**C**) and high (**F**) fibroglandular density. Data within epithelial cell (**G**) and stromal regions (**H**) of low and high fibroglandular density were evaluated using paired samples and analysed with the nonparametric Wilcoxon test (*n* = 16); results are presented as mean ± standard error of the mean (SEM). Statistical significance indicated by * for *P* < 0.05. Immunofluorescence staining for HMGB1 on low (**K**) and high (**N**) fibroglandular density. HMGB1 is showed on red channel (**J**, **M**), and DAPI nuclear counterstaining is shown in the blue channel (**I**, **L**)

To investigate potential triggers of TLR4 signalling, both endogenous and exogenous agonists were examined. HMGB1, an endogenous trigger of the TLR4 pathway, exhibited higher intensity within epithelium (*P* < 0.003***)*** and stroma (*P* < 0.001***)*** of high fibroglandular density compared to low fibroglandular density (Fig. 5). Immunofluorescence staining revealed nuclear localisation of HMGB1 in both low and high fibroglandular density regions, with high fibroglandular tissue also showing some cytoplasmic staining.

**Fig. 6:**
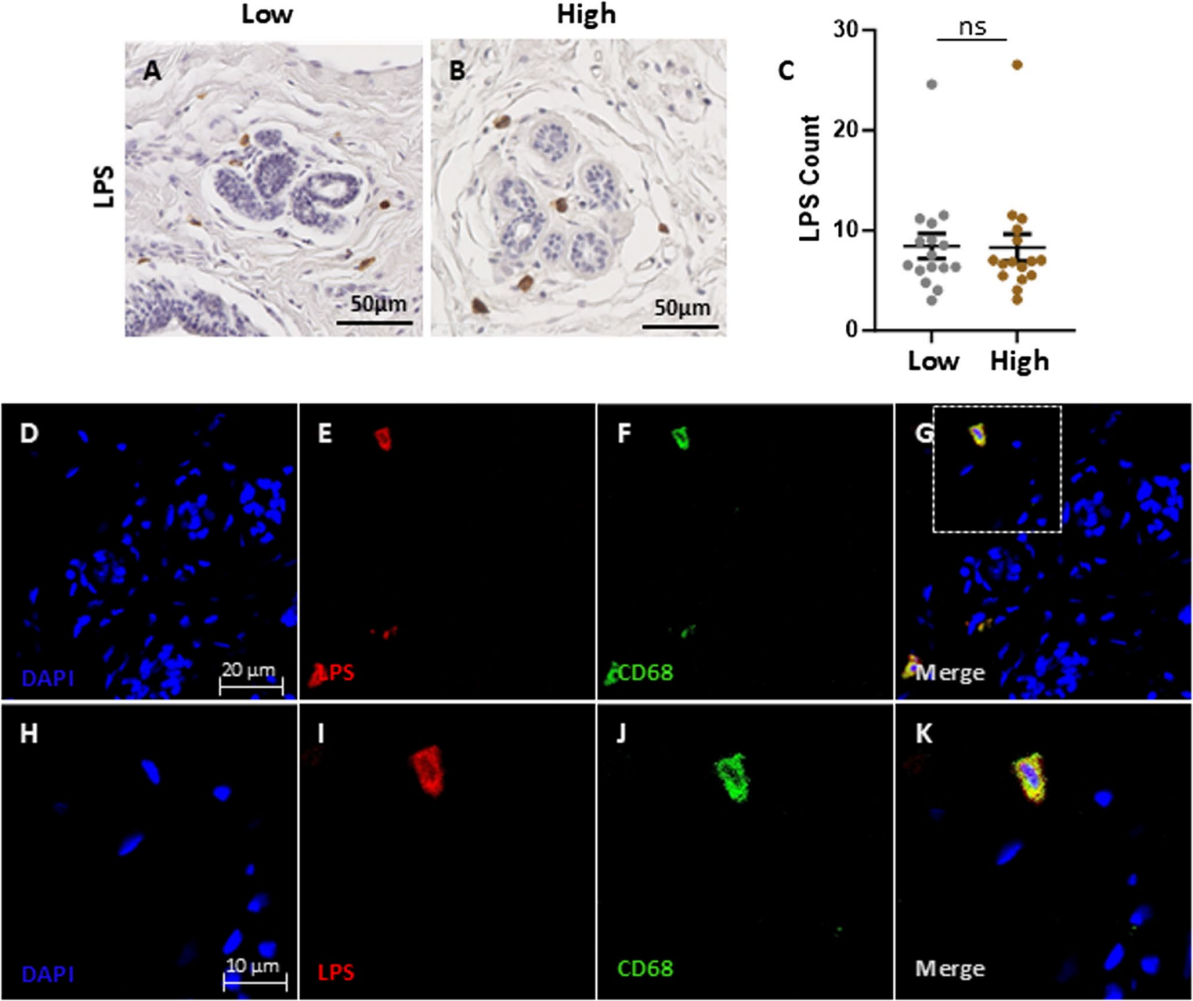
LPS abundance in high and low fibroglandular density paired breast tissue samples. Representative immunohistochemical staining of LPS from gram-negative bacteria. LPS was detected in stroma, in close approximately to the epithelium in low (**A**) and high (**B**) fibroglandular density. **C** display quantitative analysis of LPS counting, compared between low and high fibroglandular density for each sample. Data were evaluated using paired samples and analysed with the nonparametric Wilcoxon test (*n* = 16); results are presented as mean ± standard error of the mean (SEM). Statistical significance indicated by * for *P* < 0.05. Representative immunofluorescent staining of mammary glands with LPS (red channel; **E**, **I**), CD68 (green channel; **F**, **J**) and DAPI nuclear stain (blue channel; **D**, **H**). The merge image (**G**, **M**) display colocalisation of LPS and CD68. All images include both low-magnification and high-magnification views

LPS, an exogenous agonist of TLR4 and a component of gram-negative bacterial cell walls, was also assessed (Fig. 6). Variation in the presence of LPS was observed between participants, however there were no significant differences in LPS abundance between paired samples of low and high fibroglandular density (*P =* 0.9). LPS-positive cells were mostly located in the stromal compartment, and co-localisation with CD68-positive macrophages suggested phagocytosis of LPS by immune cells.

### TLR4 Agonist LPS Increases Expression of Genes Encoding the TLR4 Signalling Pathway and Inflammatory Cytokines in Mammary Epithelial Organoids

**Fig. 7:**
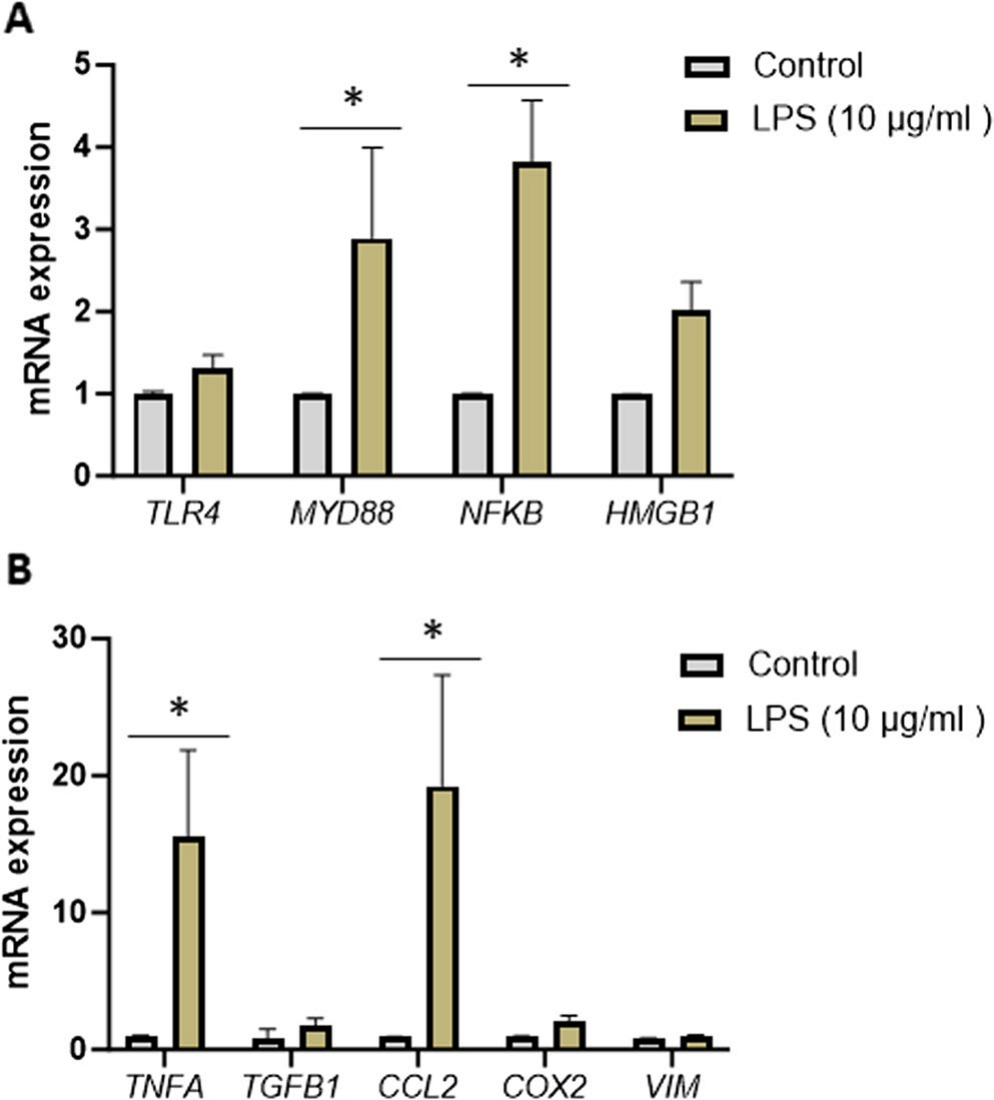
The effect of TLR4 agonist (LPS) on primary mammary epithelial organoids. **A** mRNA expression of genes encoding TLR4 signalling pathway, *TLR4*,* MYD88*,* NFKB*,* HMGB1* and (**B**) mRNA expression of genes encoding inflammatory regulators *TNFA*,* TGFB1*,* CCL2*,* COX2* and mammographic density-associated marker, *VIM* measured by RT-PCR in human mammary epithelial organoids, following treatment with 10 µg/mL of LPS for 24 h. mRNA expression normalised to beta-actin expression and presented as relative expression, where the average for the control is 1. Data were statistically analysed between control and LPS-treated groups using 2-way ANOVA with Šídák’s multiple comparisons test (*n* = 5); results are presented as mean ± standard error of the mean (SEM). Statistical significance indicated by * for *P* < 0.05

To investigate the effect of TLR4 signalling on the human mammary epithelium, 3D cultures of mammary epithelial organoids were treated with 10 µg/mL LPS for 24 h. Following treatment, mRNA expression of genes involved in TLR4 downstream signalling, inflammatory responses and mammographic density markers were evaluated. LPS stimulation upregulated the expression of genes encoding *NFKB* (*P* = 0.001), *MYD88* (*P* = 0.04), *TNFA* (*P* = 0.01) and *CCL2* (*P* = 0.001). The mRNA expression level of genes encoding *TLR4*,* HMGB1*,* TGFB1*,* COX2* and *Vimentin* were not affected by LPS treatment (Fig. 7).

## Discussion

This study suggests a key role for the TLR4 signalling pathway in the chronic inflammation observed in high mammographic density. We observed higher expression of TLR4 in both the epithelial and stromal regions of tissue with high fibroglandular density, and MYD88 and NFKB were upregulated in the epithelium. We further explored both endogenous and exogenous triggers of TLR4 signalling. HMGB1, an established endogenous danger signal and alarmin, was significantly upregulated in both epithelium and stroma of high-density tissue, with increased cytoplasmic localisation suggesting active secretion [27, 28]. To our knowledge, this is the first report linking elevated HMGB1 expression to high mammographic density, providing evidence for sterile inflammation in this context. In contrast, LPS, a bacterial endotoxin and exogenous TLR4 agonist, showed no significant difference in abundance between high and low density tissues. Additionally, stimulation of mammary epithelial cells with a TLR4 agonist induced a pro-inflammatory cytokine response, supporting the notion that TLR4 activation may contribute to the chronic inflammation associated with high mammographic density. Taken together, these findings suggest that while exogenous microbial ligands are present in breast tissue, endogenous triggers of innate immune signalling may be the more significant drivers of inflammation in high-density breast tissue.

There are well-established links between a pro-inflammatory microenvironment and cancer development [29]. Reactive oxygen and nitrogen species are created during inflammation and these chemicals can cause DNA damage increasing the risk of mutations that initiate cancer [30]. The chronic inflammatory microenvironment of breast cancer is also connected to angiogenesis, drug resistance [31], and ultimately reduced survival rate [32]. Inflammation in breast tissue may be the consequence of interactions with microbes or with dying or injured host cells [33]. The TLR family is one of the best-characterised groups of innate immune receptors involved in the generation of inflammatory responses [14]. TLR4 is present on a wide-range of cell types including macrophages, dendritic cells, B cells, endothelial cells, and epithelial cells including breast epithelial cells [34]. There is accumulating evidence that TLR4 impacts solid cancer progression including breast cancer [35]. TLR4 upregulation is related to poor survival rates and an increase in the possibility of recurrence in patients with solid cancer [36]. TLR4 can promote progression of breast cancer through regulating adhesion molecules and the invasive migration of breast carcinoma into the lymph nodes and lungs [37].

Extending previous studies suggesting a pro-inflammatory microenvironment in high mammographic density [3, 8, 13], we have found that innate immune signalling through TLR4 is upregulated in high density tissue compared to low density tissue. The upregulation of TLR4 was observed within the epithelial cell layer and macrophages in the stromal region. Within the epithelium, TLR4 was primarily observed on basal cells, which are adjacent to the stroma and fibroblasts [38]. The expansion of basal epithelial cells can be driven by stem cell plasticity in response to DNA damage [39]. The prevalence of innate immune receptors, however, is not equal on basal and luminal sides and can vary due to the different pathological conditions [40]. The higher abundance of TLR4 on basal versus luminal epithelial cells suggests that invading pathogens that have entered the ducts through the nipple are unlikely to be the predominant stimuli for TLR4 activation.

Mammographic density, characterised by highly abundant stroma, has biological similarities that resemble fibrotic-like tissue [1]. Persistent inflammation through TLR4 signalling is known to promote the development of liver fibrosis, characterised by abundant stromal components such as fibroblasts and collagen [41, 42]. The presence of upregulated TLR4 signalling in high fibroglandular density lends support for the concept that high mammographic density is a state of fibrosis driven by chronic low-level inflammation. However, mammographic density is established during puberty as the fibroglandular and adipose tissue develop, and is relatively stable throughout adult life [43]. Therefore, the ligands that initiate inflammation may also be relatively stable, suggesting that endogenous triggers of inflammation may be of greater significance than local invading pathogens.

Both an endogenous and exogenous trigger of TLR4 signalling were assessed in this study. LPS from gram-negative bacteria was present in breast tissue and located in macrophages in similar abundance in low and high fibroglandular tissue, suggesting invading pathogens are unlikely to be the primary stimuli that initiates inflammation in dense tissue. However, more investigation is required to determine and compare the microbiome in high and low mammographic density. The distinct bacterial profile may differentially interact and activate TLR4. For example, in breast cancer, bacteria that can cause DNA damage increases while the beneficial lactic acid bacteria decrease when compared to the healthy breast microbiome [44], while the bacterial count can remain steady.

On the other hand, the endogenous trigger, HMGB1, a well-known host-derived molecule generated in response to injury and inflammation [45], was increased in tissue of high fibroglandular density. It is now well-recognised that endogenous components released from stressed or damaged cells can induce sterile inflammation in the absence of invading pathogens [46]. There is a subset of endogenous triggers termed alarmins, including the HMGB1 protein, which are of special interest in sterile inflammation [47, 48]. HMGB1 is a ubiquitous protein that interacts with DNA to maintain homeostasis and regulate gene expression. When it is released to the cytoplasm, it acts as an alarmin and activates inflammatory responses. HMGB1 is released from damaged or dying cells [49], activated immune cells, and during a state of cellular stress [50]. HMGB1 is considered an important biomarker for inflammatory-based diseases. It is heightened in blood and synovial fluid and overexpressed in affected areas in a number of autoimmune diseases such as arthritis [51]. Translocated HMGB1 is linked to activated TLR4 signalling pathways and promotes cancer growth and invasion by mediating the production of inflammatory mediators [52].

Elevated expression of gamma H2A histone family member X (H2AX) in high mammographic density tissue suggests an increased occurrence of DNA damage [53], and this may be the cause of increased HMGB1 reported here. Indeed, there may be a cycle of inflammation and DNA damage in high mammographic density, with HMGB1 acting as both an outcome of DNA damage and an activator of further TLR4-mediated immune signalling. HMGB1 may impact inflammatory signalling, the abundance of epithelium, as well as stromal stiffness which are all associated with mammographic density. The stiffness of the high mammographic density microenvironment is increased because of changes in the structure and composition of its cellular components [54]. HMGB1, known as a marker for fibrosis, facilitates maintaining the fibrotic, stiff stroma by activating normal fibroblasts and promoting collagen deposition [55, 56]. HMGB1 also engages with other receptors, increasing cell proliferation [57]. Extracellular HMGB1 can bind to RAGE (Receptor for Advanced Glycation End-products) on epithelial cells and modulate cell cycle regulation by increasing cell proliferation via the phosphoinositide 3-kinase/AK transforming (PI3K/AKT) and mitogen-activated protein kinases (MAPK) pathways [58]. Furthermore, AKT provides a potent pro-survival signal, conferring resistance to apoptosis [59]. Thus, HMGB1 may be a key mediator of high mammographic density and elevated breast cancer risk, conferring a microenvironment of fibrosis, inflammation, and DNA damage. Further studies on the abundance and activation of HMGB1 in dense breast tissue are needed.

Activation of TLR4 resulted in downstream expression of genes encoding inflammatory cytokines TNFA and CCL2 in primary epithelial organoid cultures. CCL2, also known as monocytic chemotactic protein 1 (MCP1), is a chemoattractant cytokine mediating macrophage migration [60]. CCL2 is upregulated in high mammographic density breast tissue, and constitutive expression of CCL2 in a mouse model increases macrophage infiltration in the mammary gland, collagen deposition, thickness of stroma and cancer susceptibility [13]. CCL2 has been shown to have a role in the development of breast cancer by infiltrating inflammatory monocytes, abundant in the cancer microenvironment linked to cancer growth [61]. Activated fibroblasts can also boost CCL2 stimulating breast cancer stem cells and production of cytokines through STAT3 signalling pathway [62]. There is compelling evidence that blocking CCL2 and infiltration of tumour-associated macrophages can inhibit ER + breast cancer [63].

TNFA is another pro-inflammatory cytokine increased by TLR4 activation, and has known roles in autoimmunity [64], cancer [65] and fibrotic diseases [66]. TNFA has two different receptors named TNFR1 and TNFR2, when it binds to TNFR2 it initiates signalling pathway that leads to the activation of NFKB, supporting inflammation and cancer cell invasion [65]. Additionally, binding with TNFR2 is associated with increasing myofibroblast and collagen degradation which results in collagen deposition and fibrosis [67]. Consistent with this study, TNFA, among other inflammatory factors including interleukin-6, C-reactive protein, and interleukin-8, increased in tissue with a higher percentage of mammographic density in aged and waist circumference matched groups [12].

This research lends support for reducing inflammation as a potential therapeutic approach to reduce breast cancer risk [68]. Non-steroidal anti-inflammatory drugs (NSAIDs) and COX2 inhibitors are the most studied treatments targeting inflammation in breast cancer. According to meta-analysis, using NSAIDs, specifically aspirin [69] and COX2 inhibitors [70], is associated with breast cancer risk reduction. In a clinical trial, healthy postmenopausal women with high mammographic density, who received low-dose acetylsalicylic acid (ASA) showed diminished local inflammation [71]. Treatment with celecoxib, a COX2 inhibitor, before tumour induction showed a decrease in tumour growth and number, indicating the potential capability to use anti-inflammatory treatment as a prevention for women with high mammographic density and high risk of cancer development [72]. NSAIDs, especially aspirin, demonstrate potential as a preventive agent for lowering mammographic density-associated breast cancer risk [73], however large randomised controlled trials are required to properly explore this.

There is growing research interest in targeting the TLR4 signalling pathway as a potential strategy for breast cancer treatment. TLR4 overexpression is an indicator of breast tumour aggressiveness, which is expressed in 20% of immune cells recruited into the cancer microenvironment [74]. TLR4 knockdown in breast cancer cell lines decreases cancer growth by reducing inflammatory cytokine secretion [75]. Blocking NFKB increases the susceptibility of the tumour to therapy and mitigates collagen cross-linking in dense stroma [76]. MYD88 inhibitors lead to reduced progression of murine mammary malignancy and human breast cancer cell lines [77]. However, research on the use of inhibitors of TLR4 signalling to reduce mammographic density-associated breast cancer risk should consider the potential side effects of inhibiting a key pathway in innate immunity.

The limitations of this study include the restricted number of samples used in analysis, use of fibroglandular density as a surrogate for mammographic density, and LPS as a model TLR4 agonist. The ability to conduct statistical analysis on paired samples enabled the small sample size (*n* = 16 pairs) to yield significant results, as reported in similar studies [8, 13], however it is possible that a larger sample size might generate different results. This study used fibroglandular density as a surrogate marker for mammographic density. Mammographic density is a radiological measure and it can be challenging to obtain tissue samples with known mammographic density. Previous studies have reported associations between mammographic density and biological measures including the abundance of fibroglandular tissue in relation to adipose tissue which has enabled the use of fibroglandular density as a surrogate measure [1, 2, 7]. Nonetheless, there could be differences in how regions of high and low density are identified that could affect the results. Finally, use of LPS as a model TLR4 agonist is a study limitation. LPS can activate both TLR4 dependent and independent inflammatory pathways [78] and there is likely to be variability between individuals in the relative contribution of different signalling pathways in the overall inflammatory response. We have shown that the key components of the TLR4 pathway are increased in breast tissue of high fibroglandular density. However our findings are largely observational, and further TLR4 knockdown experiments are required to demonstrate that this signalling pathway is responsible for the increased inflammatory cytokines identified in the in vitro results.

## Conclusion

The TLR4 signalling pathway is upregulated in high fibroglandular density breast tissue and this is associated with increased endogenous HMGB1. Increased innate immune signalling through TLR4 may be associated with the pro-tumour inflammatory response observed in high mammographic density. TLR4 initiates its signalling pathway through MYD88 leading to the translocation of NFKB transcription factor into the nucleus and transcription of genes encoding inflammatory cytokines and chemokines, namely TNFA and CCL2. We propose that HMGB1, an endogenous trigger of TLR4, is part of a chronic cycle of DNA damage and inflammation that increases breast cancer risk associated with high mammographic density. A better understanding of the pro-inflammatory microenvironment in high mammographic density can assist in the development of novel therapeutic and prevention strategies for breast cancer.

## Data Availability

The datasets used and/or analysed during the current study are available from the corresponding author on reasonable request.
